# Impact of non-pyrethroid insecticide treated durable wall lining on age structure of malaria vectors in Muheza, Tanzania

**DOI:** 10.1186/s13104-017-3078-7

**Published:** 2017-12-19

**Authors:** Basiliana Emidi, William N. Kisinza, Franklin W. Mosha

**Affiliations:** 1 Kilimanjaro Christian Medical University College, P.O. Box 2240, Moshi, Tanzania; 2 National Institute for Medical Research, Headquarters, P.O. Box 9653, Dar Es Salaam, Tanzania; 3 National Institute for Medical Research, Amani Research Centre, P.O. Box 81, Muheza, Tanzania

**Keywords:** Non-pyrethroid treated durable wall liners, Age structure, Parous, Nulliparous, *An. funestus*, *An. gambiae s. l.*, Malaria vectors, Muheza, Tanzania

## Abstract

**Objective:**

Malaria vectors control interventions are designed to cause immediate killing or shorten mosquito lives, therefore does not allow enough time for the development of the parasites to infective stage. The wall lining is new malaria vectors control intervention in Tanzania where its impact on age structure is not well known. Therefore this study aimed at determining the impact of non-pyrethroid durable wall lining on the age structure of malaria vectors.

**Results:**

Higher proportions of *An. gambiae* sensu lato (57.1%, z = 2.66, *P* = 0.0077) and *An. funestus* (64.8%, z = 3.38, *P* = 0.001) were collected in the control clusters. Unexpectedly, significantly higher proportion of parous *An. gambiae s. l*. were collected in the intervention clusters (z = − 2.78, *P* = 0.0054). The wall lining intervention has demonstrated low impact on age structure of *An. gambiae s. l.*, this call for further studies on the efficacy of the intervention.

## Introduction

Mosquito biting behaviour changes have been reported to be induced by vector control interventions, particularly when excito-repellent insecticides are used [1]. Spraying the walls and ceiling of houses with residual insecticides tends to reduce the survival of mosquito vectors, therefore reduce malaria transmission [2]. This is because, insecticides irritancy cause high proportion of mosquitoes to exit from treated houses. However, treated walls also contribute to decreased feeding rate and resting behaviour of indoor biting mosquitoes [3]. *An. gambiae s. s*. and *An. funestus*, which are the malaria vectors, naturally prefer to feed and rests indoors [3–5]. This is in contrast to the strongly exophilic and exophagic; *An. arabiensis* [6, 7]. Nevertheless, the use of indoor chemical interventions such as long lasting insecticide treated nets (LLINs) and indoor residue spray (IRS) have reported to drive malaria vectors to feed and rest outdoors resulting in a reproductive advantage for them [4]. Such shifting would also be accompanied by feeding more outdoors at dusk or dawn rather than indoors at mid-night [4, 7, 8]. Outdoor biting mosquitoes remain as secondary sources of malaria transmission as they respond poorly to indoors insecticidal interventions [9–11].

Age grading technique is applied for establishing the parous rate of mosquito populations in order to estimate longevity [12]. Normally, mosquitoes are dissected to observe ovarian dilations in order to determine the proportions that are nulliparous (have not laid eggs) or young mosquito and parous (have laid eggs) or old mosquitoes [12, 13]. Parous mosquitoes are those that have taken a blood meal and oviposited at least once [14]. Age grading in malaria vector is important in estimating the risk of malaria in a particular area [15], with respect to the presence of interventions [16]. Currently, a novel non-pyrethroid insecticide treated durable wall liner (ITWL) which works similar to IRS has been developed. It consists of a thin sheet of cloth made from high-density polypropylene treated with a mixture of two non-pyrethroid insecticides namely; abamectin and fenpyroximate [17, 18]. The impact of ITWL intervention on age structure of malaria vectors is not well known in Tanzania. Therefore, the present study aimed at determining its impact on age structure of malaria vectors under field conditions in the study area.

## Main text

### Materials and methods

#### Study area

This cross-sectional study was conducted for the duration of 7 months from November, 2015 to May, 2016 in 18 clusters in Muheza district, North-eastern coast of Tanzania. In recent years, there have been fluctuations in the rainfall patterns with long drought periods in the study area. During implementation of this study, there were long dry seasons which accompanied with dryness of the mosquito breeding sites [19]. The district covers a geographical area of 4922 km^2^, lying between 5°S latitude and 39°E longitude. The climate is tropical, with dense rainforest over the Usambara mountain ranges and has an annual rainfall of 1000–2000 mm. Muheza district is mainly inhabited by subsistence farmers. Administratively, Muheza district is divided into six divisions comprised of 35 wards with 175 villages [20]. The area is endemic for malaria and lymphatic filariasis whereby *An. gambiae s. l.* and *An. funestus* are the main vectors of these diseases [21, 22]. *An. gambiae s. l.* in this area has also been documented to be resistant to pyrethroid insecticides [23, 24].

#### Site selection and mosquito collection

**Fig. 1:**
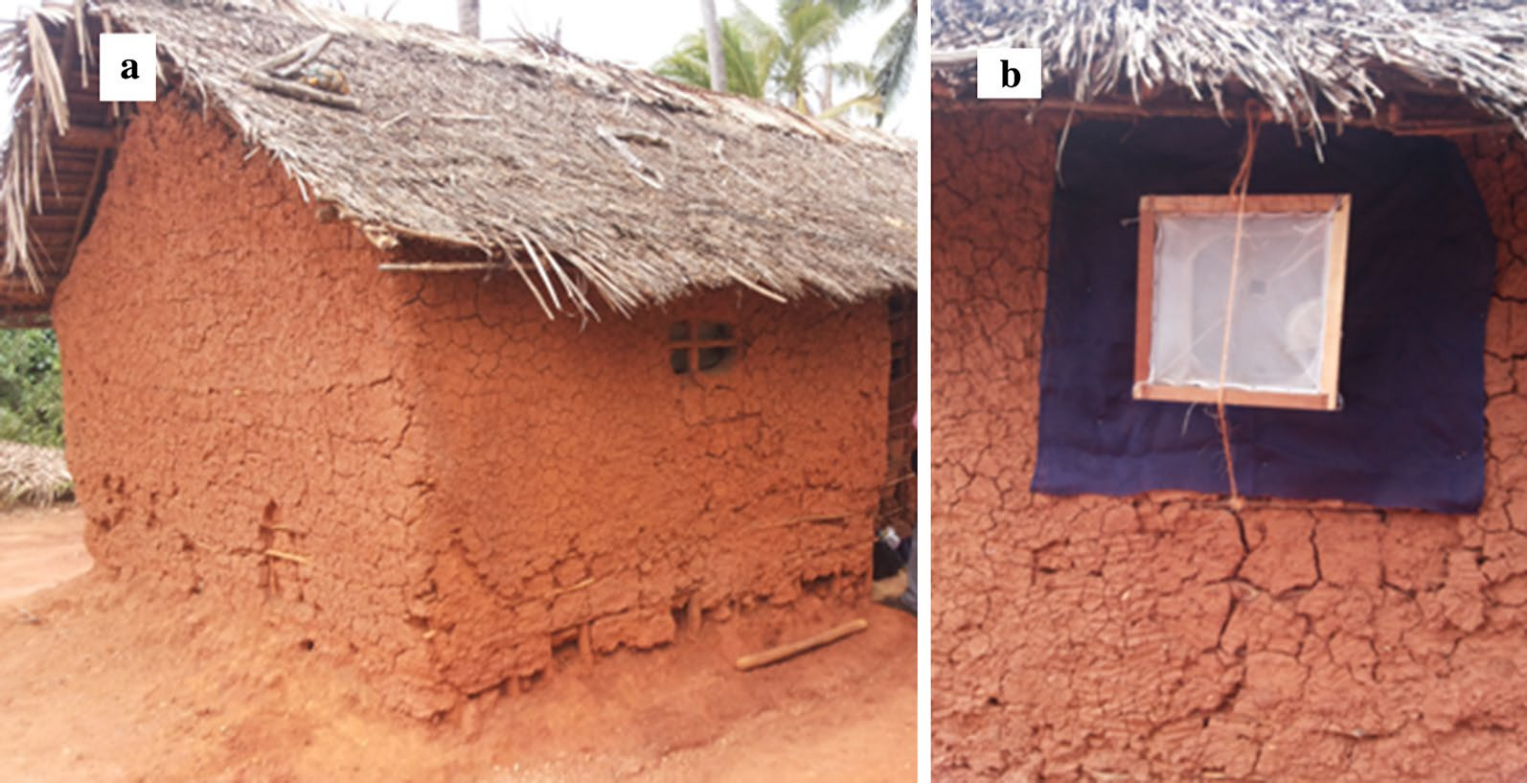
Mosquito trapping. **a** House type. **b** Exit trap set on the window

**Fig. 2:**
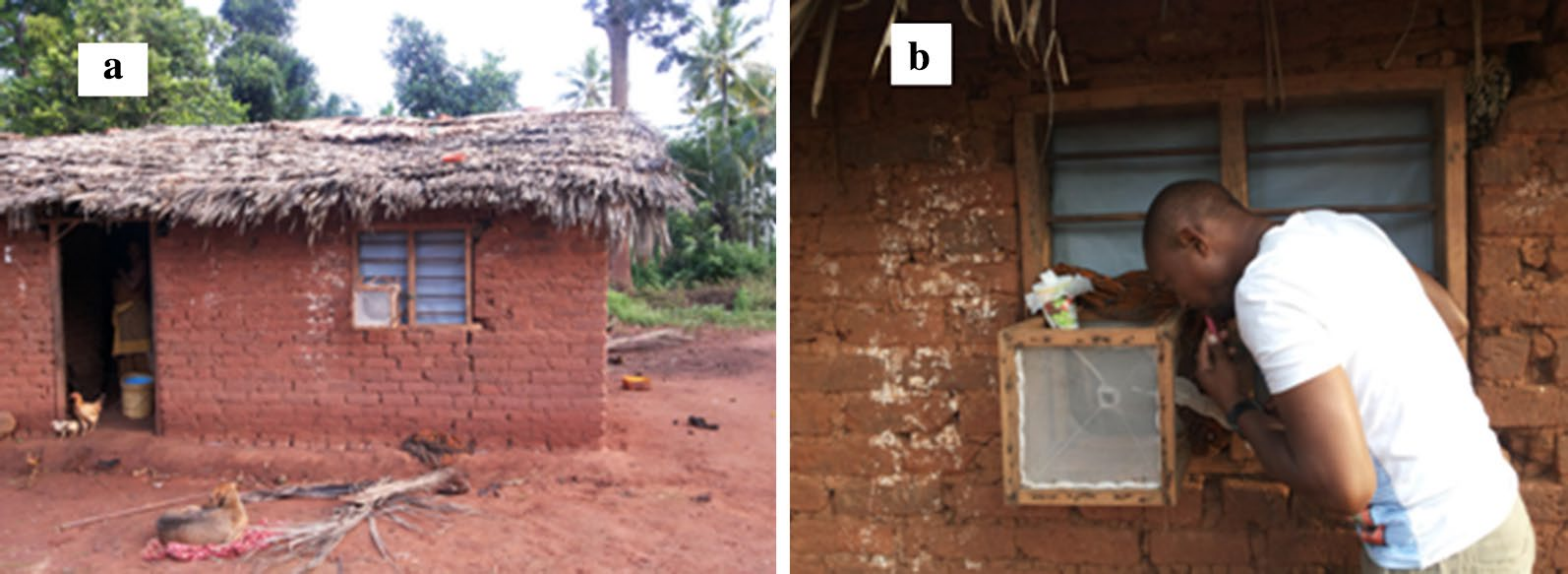
Exit trap on **a** window with ITWL, **b** collecting mosquitoes in exit trap

A total of 18 clusters were selected; nine in control clusters and nine in intervention clusters from clusters in a randomized field trial [18]. Intervention clusters were those with high percentage (over 80%) coverage of durable wall liners installation and they were provided with LLINs while control clusters were the ones which were provided with LLINs alone. The core area of each cluster had a minimum of 124 households [18]. In each cluster, four houses with open eaves and unscreened windows were selected. In each selected house only one window of the sleeping room was chosen for setting exit traps as described in the WHO [12] in order to collect mosquitoes (Figs. 1, 2).

#### Data analysis

Data were entered in excel database and transferred to Stata version 13 statistical software where a Two-sample test for proportions (z) was performed. The outcomes of interest were the proportions of parous and nulliparous among the malaria vector species and intervention arms. In all analysis a *P* value of < 0.05 was considered statistically significant.

#### Mosquito identification and dissection

Adult mosquitoes were identified morphologically using identification keys [25] for *Anopheles*, Culicines [26] and Zootaxa 700 key was used for *Aedes* mosquitoes [27]. All Culicines collected were recorded after identification and then discarded. All fresh unfed *An. gambiae s. l.* and *An. funestus* were dissected to observe ovarian dilations [12, 13]. Based on tracheolar skeins, dissected mosquitoes were categorized as parous and nulliparous [12].

#### *An. gambiae s. l.* and *An. funestus* sibling species identification

*An. gambiae s. l.* was identified to sibling species by real-time polymerase chain reaction (PCR) as described by Bass et al. [28]. *An. funestus* sibling species were identified by PCR based on species-specific primers. Electrophoresis was performed there after and amplicons were then visualized under uv-light for scoring DNA band lengths in relation to positive controls as described by Koekemoer et al. [29].

#### Malaria sporozoites determination

Malaria sporozoites determination in *An. gambiae s. l.* and *An. funestus* was done by using enzyme linked-immunosorbent assay (ELISA) technique according to Witz et al. [30] whereby mosquito head and thorax were used.

### Results

#### Mosquito collected

A total of 1757 mosquitoes were collected. Majority of mosquitoes collected were *Cx. quinquefasciatus* followed by *An. gambiae s. l.* and *An. funestus.* Malaria vectors (*An. gambiae s. l.* and *An. funestus)* accounted for 28.5% (n = 501). Majority of *An. gambiae s. l.* (57.1%, n = 205) were collected in the control clusters than in the intervention clusters and the different was statistically significant (z = 2.66, *P* = 0.0077). Similarly to *An. gambiae s. l.,* majority of *An. funestus* (64.8%, n = 92) were collected in the control clusters, and the different was statistically significant (z = 3.38, *P* = 0.001) (Table 1).

**Table 1:** Parity status of *An. gambiae s. l*. and *An. funestus* the control and Intervention arms

Mosquito species and parity status	Clusters	*An. gambiae* s. l. and A*n. funestus*, n	Proportions	95% confidence interval		z test	P value
				Lower limit	Upper limit		
Total *An. gambiae s. l.*	Control (LLINs only)	205	0.57	0.50	0.64	2.66	0.0077*
	Intervention (ITWL and LLINs)	154	0.43	0.35	0.51		
Parous *An. gambiae s. l.*	Control (LLINs only)	115	0.42	0.33	0.51	− 2.78	0.0054*
	Intervention (ITWL and LLINs)	162	0.58	0.51	0.66		
Nulliparous *An. gambiae s. l.*	Control (LLINs only)	76	0.58	0.47	0.70	1.9	0.0571
	Intervention (ITWL and LLINs)	54	0.42	0.28	0.55		
Total *An. funestus*	Control (LLINs only)	92	0.65	0.55	0.75	3.38	0.001*
	Intervention (ITWL and LLINs)	50	0.35	0.22	0.48		
Parous *An. funestus*	Control (LLINs only)	49	0.56	0.42	0.70	1.06	0.290
	Intervention (ITWL and LLINs)	39	0.44	0.29	0.60		
Nulliparous *An. funestus*	Control (LLINs only)	23	0.43	0.22	0.63	− 1.08	0.282
	Intervention (ITWL and LLINs)	31	0.57	0.40	0.75		

* Statistically significant

#### Proportions of parous and nulliparous malaria vectors in the control and intervention clusters

The proportion of parous *An. gambiae s. l*. was statistically significant higher in the intervention clusters than in the control clusters (z = − 2.78, P = 0.0054). On the other hand, the proportions of nulliparous *An. gambiae s. l*. was higher in the control clusters although not statistically significant (z = 1.9, P = 0.0571) (Table 1).

Regarding *An. funestus,* the proportion of parous was higher in the control clusters while that of nulliparous were higher in intervention clusters although not statistically significant (Table 1).

#### *An. gambiae s. l.* and *An. funestus* group sibling species composition and circumsporozoite ELISA positivity

A total of 302 *An. gambiae s. l.* and *An. funestus* group mosquitoes were identified to sibling species. Among these, *An. gambiae s. l.* accounted for 60% (n = 181) and *An. funestus* group were 40% (n = 121). PCR results has shown that, *An. gambiae s. l.* consisted of *An. gambiae s. s.* (74%, n = 134) and *An. arabiensis* (26%, n = 47). Within the *An. funestus* group, four sibling species were identified; these included; *An. funestus s. s*. (95%, n = 115), *An. leesoni* (2.5%, n = 3), *An. rivulorum* (1.7%, n = 2) and *An. parensis* (0.8%, n = 1). The circumsporozoite (CSP) ELISA results revealed that, sporozoite positive among *An. gambiae s. s.* was 5.6% while that of *An. funestus s. s.* was 2.0% with two individuals from each of the species. The overall CSP positive among *An. gambiae s. s* and *An. funestus s. s.* was 2.9%.

### Discussion

Muheza is one of the malaria endemic district in Tanzania where both *An. funestus* and *An. gambiae s. l.* are the main vectors for malaria transmission [23, 31]. Despite the fact that, the area had non-pyrethroid ITWL and LLINs, the overall sporozoite rate among *An. gambiae s. s* and *An. funestus s. s.* was 2.9 but the number of positive mosquitoes was too low to make any meaningful comparison between intervention and control clusters. This was in contrary to the previous study which was conducted in northern Orissa, India where presence of zerofly plastic sheeting interventions reduced malaria transmission and parous rate when compared to pre-intervention phase [32]. This study has shown that, in *An. gambiae s. l.,* the main sibling species were *An. gambiae s. s.* and *An. arabiensis*, similarly to a previous study [33]. Within the *An. funestus,* four sibling species identified included; *An. funestus s. s., An. leesoni, An. rivulorum* and *An. parensis* with *An. funestus s. s.* which accounted for 95%, similarly to a study by Derua et al. [21].

High proportions of *An. gambiae s. l.* and *An. funestus* in the control clusters implies that, the intervention plays a role in preventing the malaria vectors from entering the houses. Our findings concur with a study conducted in the experimental huts which found that, the presence of both LLINs and non-pyrethroid ITWL have led to a significant increase the proportions of *An. gambiae s. l.* exiting the huts [17]. This situation has implication in reduction of malaria transmission in a malaria-endemic community [34].

It was surprising to find that, statistically significantly higher proportions of parous in the intervention clusters. These findings indicate that, the new non-pyrethroid ITWL intervention has low impact on age structure of *An. gambiae s. l.* under field condition as old mosquitoes were collected in the intervention clusters and young ones in the control clusters. Our findings are consistence with a previous study conducted in the same area which showed that, both pyrethroid LLINs and the non-pyrethroid ITWL in experimental huts induced exiting among *An. gambiae s. l*. [17]. ITWL intervention does not induced mortality in *An. gambiae s. l.* as demonstrated by the present study. This could be the reason why they were able to live long enough to lay eggs in intervention clusters; a situation has implication in malaria transmission.

The proportion of parous *An. funestus* was higher in the control clusters. The present study findings concur with a previous study conducted in India, whereby the impact of vector control intervention on reduction in parity rate and house entry of *An. culicifacies* were observed in the village with olyset net compared to untreated net or no nets [35]. Similar findings have also reported from the same area that, significantly higher proportions of *An. funestus* exited through the exit trap due to the presence of pyrethroid LLINs compared to non-pyrethroid ITWL [17]. ITWL intervention has some positive practical implications as they are durable and cost effective compared to the IRS and also it improves houses in rural settings where most of the walls are made up of mud [18]. Basing on the findings from the present study, further studies to understand the efficacy of ITWL among mosquito populations are needed.

## Limitations

The study was conducted in a period of extremely dry season where the number of mosquito collected was low and this could have impact on interpretations of findings.
